# Premorbid physical activity is modestly associated with gait independence after a stroke: an exploratory study

**DOI:** 10.1186/s11556-018-0208-8

**Published:** 2018-12-26

**Authors:** Tomoko Yamaguchi, Osamu Yamamura, Tadanori Hamano, Kazuhiro Murakita, Yasunari Nakamoto

**Affiliations:** 1 Department of Community Medicine, Faculty of Medical Science, University of Fukui, Fukui, Japan; 2 Second Department of Internal Medicine, Faculty of Medical Science, University of Fukui. Fukui, Shimoaizuki 23, Matsuoka, 9101193 Japan; 3 Sakura Senju Hospital, Fukui, Japan

**Keywords:** Gait independence, Premorbid physical activities, Questionnaire, Stroke, Cerebrovascular disease

## Abstract

**Background:**

Regaining physical function after a stroke is important for independence and for performing activities of daily living. Particularly, cerebrovascular disease, which includes stroke, is not entirely avoidable. In the present study, we aimed to observe the association between premorbid physical activities and gait independence after a stroke.

**Methods:**

Consecutive cerebrovascular stroke patients were asked to fill a questionnaire regarding their premorbid physical activities. The association between gait independence at the completion of in-hospital rehabilitation and premorbid physical activities, as well as age, stroke type, lesion size, and comorbidities, was investigated statistically.

**Results:**

Of 130 consecutive patients with stroke who answered the questionnaire regarding their premorbid physical activities, 97 regained gait independence. Ambulation and occupational or household activities were most frequently performed by all the participants before stroke onset. Participants who acquired gait independence tended to indicate various premorbid activities compared to participants who did not recover gait independence. Estimating premorbid physical activities in metabolic equivalents suggested that moderate to vigorous activities were associated with an increased probability of post-stroke independent gait but this tendency was dismissed after multivariate analysis including age and history of stroke.

**Conclusions:**

Premorbid physical activity is associated with gait independence after a stroke, but this association is not as strong as those of age or history of stroke.

**Electronic supplementary material:**

The online version of this article (10.1186/s11556-018-0208-8) contains supplementary material, which is available to authorized users.

## Background

Regaining physical function after a stroke is important for patients since cerebrovascular disease (CVD) is not entirely avoidable. In 2010, CVD caused 102 million deaths and disabilities worldwide, which accounted for approximately 4.1% of all-cause mortality and disability [1]. Independent ambulation promotes independence and helps patients perform activities of daily living [2–4]. In addition, physical activity (PA) after a stroke enhances the quality of life, and regular exercise combined with abstinence from smoking lowers all-cause mortality in stroke survivors [2–5]. Thus, gait independence after a stroke has great importance for social participation, preventive medicine, as well as for rehabilitation.

Factors reported to determine or predict gait ability after a stroke include age, consciousness level at stroke onset, urinary continence, upper and lower limb function, balance, and walking ability during rehabilitation [6–9]. More recently, premorbid activities and lifestyle have been shown to be predictors of post-stroke functioning [10, 11].

Health education promotes a lifestyle that includes PA and exercise as crucial prophylaxis against arteriosclerotic disease [12–20]. In addition, moderate to vigorous PA is recently becoming known to contribute to decreasing all-cause mortality [21]. However, the direct contribution of premorbid PA to gait outcome after a stroke is not well investigated.

We carried out this exploratory study to determine the characteristics of premorbid PA in stroke patients and to ascertain whether PA has any association with gait independence after rehabilitation.

## Materials and methods

The present study was approved by the Ethics Committee of Fukui Prefectural Hospital (#11–36). Written informed consent was obtained from the patients or their relatives on enrollment into this study.

All inpatients with acute CVD introduced to the Department of Physical Medicine and Rehabilitation (PMR) of Fukui Prefectural Hospital between October 22, 2011 and March 31, 2013, were considered for inclusion. Patients who had suffered from ischemic and hemorrhagic strokes were included. Patients with intracranial tumors, traumatic brain injuries, subarachnoid hemorrhages due to ruptured aneurysms, or chronic subdural hematomas were excluded, as well as were pediatric patients. Standardized treatments were performed by board-certified neurologists or neurosurgeons at the time of hospitalization for all the patients.

The rehabilitation therapists in charge asked the participants to complete a questionnaire regarding their pre-stroke exercise routine and daily PA at the beginning of the rehabilitation therapy (Additional file 1). The questionnaire was prepared in accordance with the Japanese Kenko Nippon 21 Exercise Guide [22], in which the intensities of exercises and daily activities were estimated in metabolic equivalents (METs). For example, watching television in a sitting position, performing light household activities such as pressing clothes, walking at a speed of 67 m/min on a flat surface, and climbing stairs have intensities of 1, 2.3, 3, and 8 METs, respectively [22, 23]. The participants were asked to indicate all the activities they had performed for more than 1 h per week during the month prior to their admission. The therapists helped the participants complete the questionnaire if they needed assistance because of seeing or hearing difficulties; when the participants had cognitive impairment, their relatives were asked to fill the questionnaire. The participants’ maximal daily PAs were classified into integral METs, from 1 to 8 METs.

Basic information, such as sex, age, and type of disease, was gathered from hospital records, along with the following information: date on which the rehabilitation therapy was started; coexistence of aphasia, agnosia, or apraxia; ongoing or past treatment of diabetes mellitus, hypertension, hyperlipidemia, cardiac disease, malignancy, orthopedic complication, and psychiatric disease; smoking history, and CVD history. Stroke size was represented as the maximal diameter of the affected region measured on cross-sectional images of the initial computed tomography or magnetic resonance imaging findings. The measurement was performed under the supervision of a board-certified radiologist. Paresis was assessed according to the Brunnstrom Recovery Stage (BRS). Sensory disturbance was categorized as “no symptom/slight numbness” or “overt disturbance.”

When participants completed their inpatient rehabilitation program, gait independence was evaluated using the Functional Independence Measure (FIM), in which the activities of daily living of the patients were classified into seven levels according to their ability. Levels 1 and 2 indicated dependence on others, levels 3–5 indicated the need for varying degrees of assistance, and levels 6 and 7 indicated independence [24]. A physiatrist who was blinded to the premorbid PA level evaluated gait independence to avoid bias in the rehabilitation goal setting. The participants were then assigned to one of two categories according to their gait independence as follows: “walking alone” (WA) group, which included participants with FIM levels 6 and 7, and “not walking alone” (NWA) group, which included participants with FIM levels 5 and below.

Dementia was evaluated using the Japanese domestic scale presented by the Ministry of Health, Labor and Welfare, which is mainly used in the Long-term Care Insurance System in Japan. This scale focuses on how much service a patient/client needs, rather than on psychopathological severity [25]. The classes are as follows: class 0, no sign of dementia; class 1, signs of dementia, e.g. forgetfulness but an independent daily life; class 2, daily life involves surveillance because of signs of dementia, difficulty in communication, and differences in behavior; class 3, daily life is dependent on others because of the above symptoms; class 4, continuous help is needed from others because of the above symptoms; and class M, treatment is needed because of psychiatric, behavioral, or physical symptoms. In the present study, those in classes ≥2 were defined as having overt dementia.

Overlapping of coexisting diseases has been reported to be a factor of stroke outcome deterioration [11, 26]. The comorbidity index (CI) was defined as the total number of the following risk factors involved: hypertension, diabetes mellitus, hyperlipidemia, cardiac disease, malignancy, smoking history, and overt dementia.

Anonymous patient records were created in FileMaker Pro 8.5 ver. 2 (FileMaker Inc., Santa Clara, CA, USA), the data were calculated using Excel for Mac 2004 ver. 11.6.5 (Microsoft, Redmond, WA, USA) and transferred to StatView ver. 5 (SAS Institute, Cary, NC, USA) for statistical analysis.

### Statistical analysis

Student’s t-test was used for participants’ ages, hospital stay length, and stroke size. Data are presented as mean ± SD.

Chi-squared test for independence was used to check for correlations between gait independence and the following factors: each daily activity, coexisting disease, and stroke type. As we found a possible tendency that participants with hemorrhagic stroke with ventricular perforation (HSVP) recovered poorly compared to those with other types of stroke, an inter-disease comparison was performed between HSVP and the other types of stroke.

Mann-Whitney U-test was used to determine the association between gait outcome and CI or maximal daily PA.

Logistic regression analysis was used to ascertain how increasing intensities of PA contributed to gait independence. The odds represented the contribution of premorbid PA to gait independence as it increased by 1 MET from a lower intensity.

Multiple logistic regression analysis was performed to identify the factors that contributed the most to gait independence. Possible explanatory variables; namely, age, CVD history, and premorbid PA were added to the analyses to check for confounding factors. Stroke size or the type of disease were not used as explanatory variables, because we were unsure whether premorbid PA has any effect on them, as vigorous PA might have prophylactic effects on arteriosclerotic disease [12, 17, 19]. CI and dementia were not used as explanatory variables either, because onset and treatment history of comorbidities were not confirmed.

A *p* - value of < 0.05 was considered statistically significant; Bonferroni correction was used for multiple analyses.

## Results

### Participants’ baseline characteristics

One hundred and thirty participants (86 men and 44 women) were enrolled. Their mean age was 71.7 ± 10.4 years. Therapist-led rehabilitation began after a mean period of 2.57 ± 1.21 days from admission.

Length of hospital stay was 48.1 ± 51.8 days. The diagnosis was lacunar infarction in 26 participants, non-lacunar infarction in 69, HSVP in 10, and hemorrhagic stroke without ventricular perforation in 25 patients.

A majority of the participants had light paresis (BRS 5 or 6), no sensory disturbance or slight numbness, two or three comorbidities, and no dementia. The details of the participants are shown in Table 1 and Additional file 2: Table S1.

**Table 1:** Characteristics of the participants

	WA group	NWA group	*p* - value
All participants (*n* = 130)	97	33	
Age ± SD (years)	69.3 ± 10.1	79.0 ± 7.9	< 0.0001
Sex (male / female)	64 / 33	22 / 11	> 0.9999
Hospital stay length (days)	41.9 ± 45.0	66.3 ± 65.4	0.0188
Stroke type			
HS with ventricular perforation	4 (4.1)	6 (18.2)	reference^§^
HS without ventricular perforation	20 (20.6)	5 (15.2)	0.0560
IS, lacunar	21 (21.6)	5 (15.2)	0.0483
IS, non-lacunar	52 (53.6)	17 (51.5)	0.0533
Stroke size (mm)	21.4 ± 17.9	21.3 ± 15.7	0.9759
Comorbidities			
CVD history	19 (19.6)	18 (54.5)	0.0003
Dementia	9 (9.3)	14 (42.4)	< 0.0001
DM	30 (30.9)	14 (42.4)	0.3209
HT	70 (72.2)	26 (78.8)	0.6041
HLP	26 (26.8)	7 (21.2)	0.6847
Smoking	47 (48.5)	10 (30.3)	0.1070
Cardiac disease	30 (30.9)	17 (51.5)	0.0553
Malignancy *	8 (8.2)	5 (15.2)	0.4202
CI			0.1016
Mode	2	3	
Median	2	3	
Maximal daily activity (METs)			0.0046
Mode	8	8	
Median	8	5	

*WA* walk alone, *NWA* not walk alone, *SD* standard deviation, *HS* hemorrhagic stroke, *IS* ischemic stroke, *CVD* cerebrovascular disease, *DM* diabetes mellitus, *HT* hypertension, *HLP* hyperlipidemia, *MET* metabolic equivalent, *CI* Comorbidity index (see text), *METs* metabolic equivalents

*t*-test: age, size, hospital stay length. Chi-squared test: sex, type of disease, comorbidities. Mann-Whitney U-test: CI, maximal daily activity

§: Comparison was made between HSVP and other stroke types

* Data was missing for a participant in the WA group

Data are shown separately according to gait independence

Gait outcome at the completion of the in-hospital rehabilitation was FIM level 7 in 80 participants, level 6 in 17, level 5 in 21, level 2 in 1, and level 1 in 11 participants. No participant had FIM level 3 or 4. Of the 130 participants, 97 were assigned to the WA group and 33 were assigned to the NWA group.

### Premorbid PA in general

The most frequently indicated premorbid PAs were walking at various speeds, watching television, housecleaning, climbing stairs, and driving a car or scooter (Additional file 3: Table S2). Ambulation and occupational or household activities were more frequently indicated than sports or recreational activities.

**Fig. 1:**
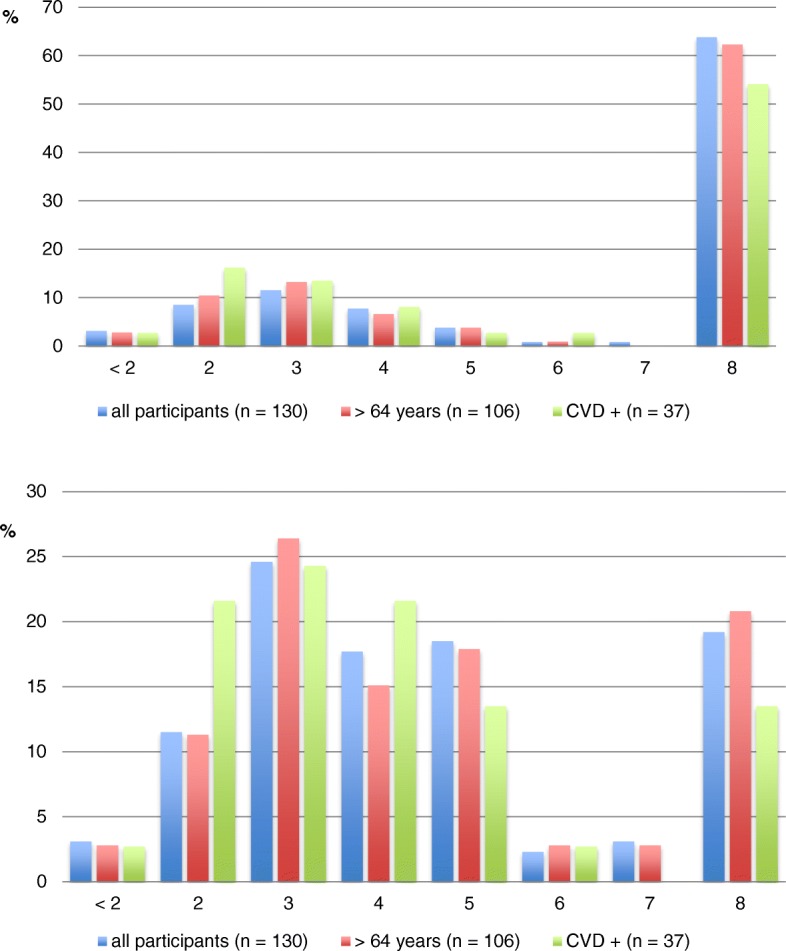
Premorbid maximal physical activity in METs. Above: including all activities. Below: excluding stair-climbing. Participants are presented in percentages (%). CVD: cerebrovascular disease; CVD +: participants with cerebrovascular disease; METs: metabolic equivalents

As shown in Fig.1, more than half of the participants had done PAs ≥ 8 METs regardless of age or CVD history. Excluding stair-climbing, the most relevant PA intensities were of 8 and 3 to 5 METs; reflecting that many of the participants had indicated farming, household jobs, and ambulation. In a subgroup of patients with a history of CVD, however, more than one-fifth of the participants had indicated 2 METs as maximal PA, which is lower than walking on a flat surface.

### Comparison between the WA and NWA groups

Participants in the NWA group were significantly older than those in the WA group (*p* <  0.0001, Table 1). Participants with HSVP tended to scarcely recover gait independence compared to participants with other types of stroke. The frequency of CVD history (*p* = 0.0003) and overt dementia (*p* <  0.0001) were significantly higher in the NWA group. Cardiac disease tended to be observed more frequently in the NWA group (*p* = 0.0533). Other comorbidities were similar between the two groups. Participants in the NWA group stayed in the hospital longer than those in the WA group (*p* = 0.0188).

Generally, participants in the WA group tended to take part more in various activities (Additional file 3: Table S2) than those in the NWA group. Statistically, washing clothes with a machine (*p* = 0.0441), driving (*p* = 0.0017), and mowing/snow removing (*p* = 0.0333) were the activities which more participants in the WA group indicated. The mode of daily PA intensity was 8 METs in both groups, and the median was 8 METs in the WA group and 5 METs in the NWA group. Premorbid PA intensity was significantly lower in the NWA group (*p* = 0.0046, Table 1).

Maximal daily PA ≥ 4 METs seemed to improve gait independence after a stroke (Table 2). However, multiple logistic regression analysis including age, CVD history, and premorbid PA, suggested a statistical significance for age (*p* = 0.0002) and CVD history (*p* = 0.0041) but not for premorbid PA ≥ 4 METs (*p* = 0.1103) or any higher METs (*p* – values not shown).

**Table 2:** Logistic regression analyses for maximal daily activity and walk independence among all the participants

	*p* - value	odds ratio	95% C.I.
Maximal daily activity			
≥ 2 METs	0.2728	3.065	0.414–22.681
≥ 3 METs	0.1206	2.472	0.788–7.752
≥ 4 METs	0.0032	3.730	1.556–8.941
≥ 5 METs	0.0370	2.400	1.054–5.463
≥ 6 METs	0.0201	2.618	1.163–5.896
≥ 7 METs	0.0064	3.111	1.375–7.040
≥ 8 METs	0.0090	2.957	1.310–6.674

*MET*, metabolic equivalents, *C.I.* confidence interval

Since age and CVD history seemed to have a great influence on gait independence, we attempted to stratify the participants according to these factors.

All participants ≤63 years old regained gait independence, therefore we reanalyzed the 106 participants who were ≥ 64 years old; 73 were assigned to the WA group and 33 to the NWA group. Participants in the WA group were significantly younger (73.6 ± 6.5 vs. 79.0 ± 7.9 years, *p* = 0.0003), with fewer participants having a history of CVD (18 vs. 13, *p* = 0.0003). CI tended to be higher in the NWA group (median 3, mode 3) than in the WA group (median 2, mode 2, *p* = 0.0555). Participants in the WA group indicated higher premorbid PA than those in the NWA group; median 8 vs. 5 METs and mode 8 vs. 8 METs (*p* = 0.0100). The odds ratio for gait independence increased as premorbid PA ≥ 4 METs increased in intensity (Table 3). However, multiple logistic regression analyses including age, CVD history, and premorbid PA, showed statistical significance for age (*p* = 0.0100) and CVD history (*p* = 0.0031) but not for premorbid PA ≥ 4 METs (*p* = 0.1223) or any higher METs (*p* – values not shown*)*.

**Table 3:** Logistic regression analyses for maximal daily activity and walk independence among ≥64-year-old participants

	*p* - value	odds ratio	95% C.I.
Maximal daily activity			
≥ 2 METs	0.2168	4.645	0.406–53.152
≥ 3 METs	0.2188	2.095	0.645–6.811
≥ 4 METs	0.0087	3.401	1.363–8.487
≥ 5 METs	0.0499	2.368	1.000–5.608
≥ 6 METs	0.0257	2.631	1.124–6.157
≥ 8 METs	0.0123	2.971	1.267–6.968

*MET* metabolic equivalent, *C.I.* confidence interval, No participant indicated 7 METs

Thirty-seven participants had a history of stroke; 19 of them were assigned to the WA group and 18 to the NWA group. Participants in the WA group were significantly younger than the others (68.2 ± 10.7 vs. 80.6 ± 6.6 years, *p* = 0.0002). CI was similar between both groups; the median was 2 in the WA group and 2.5 in the NWA group, and mode was 2 in both groups (*p* = 0.1435). Premorbid PA intensity was significantly lower in the NWA group (median 5, mode 8) than in the WA group (median 8, mode 8; *p* = 0.0209). The odds ratio for gait independence increased as premorbid PA ≥ 4 METs increased in intensity (Additional file 4: Table S3), although this result must be interpreted carefully considering the small number of participants. Multiple logistic regression analyses including age and premorbid PA suggested that age was a significant factor (*p* = 0.0068) but not premorbid PA ≥ 4 METs (*p* = 0.5912) or any higher METs (*p* - values not shown*)*.

## Discussion

The results of the present study suggest several associations of premorbid PA with gait independence after stroke. First, the participants who regained gait independence tended to have performed more PAs, whether mild or vigorous. In addition, we determined the threshold, premorbid PA ≥ 4 METs seemed to be associated with gait independence.

PA ≥ 4 METs may have had physical training effects on the muscular fitness, balance, and cardiopulmonary fitness of the participants. Leg strength was one of the determinants of gait ability among stroke survivors [27]. Additionally, as recently reported, good balance after a stroke is necessary for better gait recovery [8, 9]. Gait after a stroke requires increased energy owing to spasticity and to compensate for affected limbs [28]. As Kelly et al. reported [29], aerobic capacity declines several weeks after a stroke and the disturbed cardiopulmonary fitness affects gait performance in the subacute phase. Participants who engaged in vigorous PAs before stroke onset might have had a better physical condition to overcome the dysfunction described above, and recover favorably during the rehabilitation period. The duration of this additional effect is unknown but usually, rehabilitation begins soon enough. The majority of PAs ≥ 4 METs included ambulation and occupational or household activities. These activities do not require sports facilities or expensive equipment, nor are they gender-restricted; consequently, they can be performed with the least barrier.

Mild PA, namely, some household jobs and driving were indicated more frequently by participants who regained gait independence. These PAs tend to reduce sedentary time, lead to participation in social activities, and contribute to better cognitive function. Reducing sedentary time is known to improve health and life prognosis [30]. In addition, participation in social activities is reported to be useful for better cognition to some degree [31–33]. Dementia was an unfavorable factor for functional outcomes after a stroke [34–36].

Age and CVD history were the strongest unfavorable factors for gait independence. To determine whether the involvement of old participants or stroke survivors in vigorous PAs contributes to gait independence, further cohort studies will be needed.

This study has some limitations. First, not all stroke patients were included; patients without neurological deficits are often discharged without rehabilitation therapy, and patients in a life-threatening condition are sometimes not introduced to the PMR department. In addition, as the study included the use of questionnaires, relatively cooperative participants might have represented the majority. Thus, the pathological influence of premorbid PA might have been under- or overestimated owing to these selection biases.

Second, precise evaluation of PA was difficult. As noted by Ainsworth et al., a compendium to estimate PA in METs has not been developed, to determine the precise energy cost of each PA [23]. The amount of PA was also not examined in this study. Again, we could not include all the activities that the participants had performed. A quantitative study using instruments (e.g. accelerometer) will overcome these deficiencies.

Third, the small sample size made the subgroup analyses difficult.

Finally, the interpretation of the results was ambiguous, because PA plays a part as the modifier of comorbidity and arteriosclerotic disease. In the present study, we hypothesized that premorbid PA was an etiological factor. Stroke size and severity, comorbidity, and dementia were treated as response variables in the present study, although they have often been treated as explanatory variables for gait outcome. Similarly, the symptoms observed post-stroke onset were not used as explanatory variables, although they were often used in that way in cross-sectional studies [6–9].

## Conclusion

In conclusion, premorbid PA was modestly associated with gait independence after stroke. Ambulation and occupational or household activities were most frequently performed before stroke onset. Premorbid PA ≥ 4 METs was associated with an increased likelihood of gait independence, although this effect was not as strong as those of age and CVD history.

## Additional files

Additional file 1: Questionnaire given to participants (converted to English). (PDF 30 kb)

Additional file 2: **Table S1.** Details of the participants. Description of data: Data are shown separately according to gait independence. (PDF 161 kb)

Additional file 3: **Table S2.** Activities asked in the questionnaire and the number of patients who indicated for each activity. Physical activities are indicated in increasing intensity (METs) from watching television (1 MET) to farming, stockbreeding, and fishing (8 METs). (PDF 207 kb)

Additional file 4: **Table S3.** Logistic regression analyses of maximal daily activity and walk independence among the participants with a history of cerebrovascular disease. Daily activities are presented from those of ≥2 METs to ≥8 METs. No participants indicated 7 METs. (PDF 179 kb)

## Abbreviations

BRS: Brunnstrom recovery stage
CI: Comorbidity index.
CVD: Cerebrovascular disease
FIM: Functional Independence Measure
HSVP: Hemorrhagic stroke with ventricular perforation
MET: Metabolic equivalent
NWA: Not walking alone (see text)
PA: Physical activity
WA: Walking alone (see text)
