# Supplementary material for: Premorbid physical activity is modestly associated with gait independence after a stroke: an exploratory study
Source: Eur Rev Aging Phys Act. 2018 Dec 26;15:18. doi: 10.1186/s11556-018-0208-8 (PMC6305997; doi:10.1186/s11556-018-0208-8)
Supplement: Supplementary file 1 — Questionnaire given to participants (converted to English). (PDF 30 kb) [file 11556_2018_208_MOESM1_ESM.pdf]

Additional file

Questionnaire given to participants (converted to English)

Please check all daily (≥ 1 hr / week) activities and exercises you have done shortly (approximately within 1 month) before hospital admission.

- ☐ watching TV
- ☐ listening to music
- ☐ reading
- ☐ desk work
- ☐ handcrafting
- ☐ cooking
- ☐ washing clothes using a machine
- ☐ pressing clothes
- ☐ child care
- ☐ walking with a carriage
- ☐ yoga
- ☐ washing dishes
- ☐ housecleaning (light)
- ☐ driving a car or scooter
- ☐ stretching
- ☐ playing a musical instrument
- ☐ walking (slowly)
- ☐ bowling, playing volleyball
- ☐ weight-training (light)
- ☐ walking a dog
- ☐ carpentry
- ☐ housecleaning (moderate)
- ☐ packing
- ☐ exercising at home
- ☐ playing golf (using a cart)
- ☐ walking (moderate)
- ☐ cycling
- ☐ aquatic exercise
- ☐ walking (intense)
- ☐ playing golf (on foot)
- ☐ mowing, removing snow
- ☐ dancing
- ☐ playing baseball, soccer
- ☐ moving furniture
- ☐ running, swimming, mountain-climbing
- ☐ climbing stairs
- ☐ farming, stockbreeding, fishing
- ☐ others (please list other activities and exercises here):
