# Supplementary material for: Premorbid physical activity is modestly associated with gait independence after a stroke: an exploratory study
Source: Eur Rev Aging Phys Act. 2018 Dec 26;15:18. doi: 10.1186/s11556-018-0208-8 (PMC6305997; doi:10.1186/s11556-018-0208-8)
Supplement: Supplementary file 2 — Table S1. Details of the participants. Description of data: Data are shown separately according to gait independence. (PDF 161 kb) [file 11556_2018_208_MOESM2_ESM.pdf]

Supplementary Table 1: Details of the participants

	WA group	NWA group
Number	97 (100 %)	33 (100 %)
Age (years old)		
< 64	24 (24.7)	0 (0)
64 - 69	26 (26.8)	4 (12.1)
70 - 79	33 (34.0)	12 (36.4)
80 - 89	14 (14.4)	16 (48.5)
90 <	0 (0)	1 (3.0)
Paresis		
BRS 1 – 2	0 (0)	6 (18.2)
BRS 3 – 4	6 (6.2)	4 (12.1)
BRS 5 – 6	91 (93.8)	23 (69.7)
Sensory disturbance		
none or slight	90 (92.8)	26 (78.8)
overt	4 (4.1)	5 (15.2)
unknown	3 (3.1)	2 (6.1)
Dementia		
class 0	79 (81.4)	15 (45.5)
class 1	9 (9.3)	4 (12.1)
class 2	6 (6.2)	4 (12.1)
class 3	3 (3.1)	3 (9.1)
class 4	0 (0)	6 (18.2)
class M	0 (0)	1 (3.0)

CI		
0	6 (6.2)	0 (0)
1	19 (19.6)	4 (12.1)
2	34 (35.1)	10 (30.3)
3	24 (24.7)	11(33.3)
4	12 (12.4)	6 (18.2)
5	1 (1.0)	0 (0)
6	1 (1.0)	2 (6.1)
Premorbid PA (METs)		
1	2 (2.1)	2 (6.1)
2	6 (6.2)	4 (12.1)
3	7 (7.2)	8 (24.2)
4	9 (9.3)	1 (3.0)
5	3 (3.1)	2 (6.1)
6	0 (0)	1 (3.0)
7	1 (1.0)	0 (0)
8	69 (71.1)	15 (45.5)

WA: walk alone; NWA: not walk alone; MET: metabolic equivalent; CI: comorbidity index (see text)

Data are shown separately according to gait independence.
