# Supplementary material for: Premorbid physical activity is modestly associated with gait independence after a stroke: an exploratory study
Source: Eur Rev Aging Phys Act. 2018 Dec 26;15:18. doi: 10.1186/s11556-018-0208-8 (PMC6305997; doi:10.1186/s11556-018-0208-8)
Supplement: Supplementary file 3 — Table S2. Activities asked in the questionnaire and the number of patients who indicated for each activity. Physical activities are indicated in increasing intensity (METs) from watching television (1 MET) to farming, stockbreeding, and fishing (8 METs). (PDF 207 kb) [file 11556_2018_208_MOESM3_ESM.pdf]

Supplementary Table 2: Activities asked in the questionnaire and the number of patients who indicated for each activity

Activity	Approximate METs	WA (%) (N = 97)	NWA (%) (N = 33)	*p* - value
Watching television	1	87 (89.7)	29 (87.9)	> 0.9999
Listening to music	1	31 (32.0)	10 (30.3)	> 0.9999
Reading	1.3	47 (48.5)	12 (36.4)	0.3161
Desk work	1.5-1.8	34 (35.1)	6 (18.2)	0.1106
Hand craft	1.5-1.8	6 (6.2)	2 (6.1)	> 0.9999
Cooking	2	47 (48.5)	10 (30.3)	0.1070
Washing clothes using machine	2	51 (52.6)	10 (30.3)	0.0441
Pressing clothes	2.3	17 (17.5)	2 (6.1)	0.1851
Child care	2.5	10 (10.3)	1 (3.0)	0.3494
Walking with a carriage	2.5	3 (3.1)	0 (0)	0.7256
Yoga	2.5	3 (3.1)	0 (0)	0.7256
Washing dishes	2.5	51 (52.6)	14 (42.4)	0.4202
Housecleaning (light)	2.5	55 (56.7)	17 (51.5)	0.7528
Driving a car or scooter	2.5	59 (60.8)	9 (27.3)	0.0017
Stretching	2.5	11 (11.3)	3 (9.1)	0.9721
Playing a musical instrument	3	1 (1.0)	0 (0)	> 0.9999
Walking (slowly)	3	54 (55.7)	14 (42.4)	0.2652
Bowling, playing volley ball	3	2 (2.1)	0 (0)	0.9900
Weight training (light)	3	4 (4.1)	0 (0)	0.5476
Walking a dog	3	7 (7.2)	1 (3.0)	0.6563
Carpentry	3-3.5	10 (10.3)	3 (9.1)	> 0.9999
Housecleaning (moderate)	3.5	34 (35.1)	6 (18.2)	0.1106
Packing	3.5	5 (5.2)	1 (3.0)	0.9823
Exercising at home	3.5	24 (24.7)	8 (24.2)	> 0.9999
Playing golf (using a cart)	3.5	6 (6.2)	2 (6.1)	> 0.9999
Walking (moderate)	3.8	42 (43.3)	13 (39.4)	0.8507

Cycling	4	27 (27.8)	6 (18.2)	0.3848
Aquatic exercise	4	2 (2.1)	0 (0)	0.9900
Walking (intense)	4-5	11 (11.3)	2 (6.1)	0.5910
Playing golf (on foot)	4.5	1 (1.0)	0 (0)	> 0.9999
Mowing, removing snow	4.5-6	36 (37.1)	5 (15.2)	0.0333
Dancing	4.8 – 6	5 (5.2)	0 (0)	0.4202
Playing baseball, soccer	5 -7	1 (1.0)	0 (0)	> 0.9999
Moving furniture	6	6 (6.2)	1 (3.0)	0.8047
Running, swimming, mountain climbing	6-11	2 (2.1)	0 (0)	0.9900
Climbing stairs	8	57 (58.8)	15 (45.5)	0.2603
Farming, stock breeding, fishing	8	20 (20.6)	4 (12.1)	0.4082

WA: patients who regained independent gait. NWA: patients who did not regain independent gait. METs: metabolic equivalents. Chi-squared tests were performed for each activity and for gait independence.
