# Supplementary material for: Premorbid physical activity is modestly associated with gait independence after a stroke: an exploratory study
Source: Eur Rev Aging Phys Act. 2018 Dec 26;15:18. doi: 10.1186/s11556-018-0208-8 (PMC6305997; doi:10.1186/s11556-018-0208-8)
Supplement: Supplementary file 4 — Table S3. Logistic regression analyses of maximal daily activity and walk independence among the participants with a history of cerebrovascular disease. Daily activities are presented from those of ≥2 METs to ≥8 METs. No participants indicated 7 METs. (PDF 179 kb) [file 11556_2018_208_MOESM4_ESM.pdf]

Supplementary Table 3: Logistic regression analyses of maximal daily activity and walk independence among the participants with a history of cerebrovascular disease

	p - value	odds ratio	95% C.I.
Maximal daily activity			
≥ 2 METs	0.9780	222818.423	0.000 –
≥ 3 METs	0.3443	2.429	0.386 – 15.277
≥ 4 METs	0.0332	5.333	1.142 – 24.903
≥ 5 METs	0.0754	3.500	0.880 – 13.927
≥ 6 METs	0.0371	4.400	1.092 – 17.722
≥ 8 METs	0.0170	5.600	1.360 – 23.062

METs: metabolic equivalents; C.I.: confidence interval. No participants indicated 7 METs.
